# NEK6 Knockout Causes Defects in Mitochondrial Morphology and Respiration

**DOI:** 10.3390/cells15100924

**Published:** 2026-05-18

**Authors:** Fernando Riback da Silva, Pedro Rafael Firmino Dias, Isadora Carolina Betim Pavan, Andressa Peres de Oliveira, Fernanda Luisa Basei, Leticia Ester dos Santos, Lizandra Maia de Sousa, Sílvio Roberto Consonni, André Gustavo de Oliveira, Leonardo Reis Silveira, Jörg Kobarg

**Affiliations:** 1 Faculty of Pharmaceutical Sciences, University of Campinas (UNICAMP), Campinas 13083-970, SP, Brazilpedrofirminodias@gmail.com (P.R.F.D.); isadora.bpavan@gmail.com (I.C.B.P.);; 2 Department of Biochemistry and Tissue Biology, Institute of Biology, University of Campinas (UNICAMP), Campinas 13083-970, SP, Brazil; 3 Obesity and Comorbidities Research Center (OCRC), Department of Structural and Functional Biology, Institute of Biology, University of Campinas (UNICAMP), Campinas 13083-970, SP, Brazil

**Keywords:** nek kinases, mitochondrial respiration, mitochondrial function, CRISPR-cas9 gene knockout

## Abstract

**Highlights:**

**What are the main findings?**

- NEK6 gene knockout in the DU–145 prostate cancer cell line shows impaired mitochondrial respiration and a mitochondrial fragmentation phenotype.
- Furthermore, the knockout cells show decreased levels of mitochondrial mass markers, altered expression of mitochondrial fusion/fission regulators, increased autophagy markers, and an increase in endoplasmic reticulum–mitochondria contacts.

**What are the implications of the main findings?**

- Our findings suggest a new role of NEK6 in the regulation of mitochondrial homeostasis, especially respiration and mitochondrial ultrastructure.
- The secondary consequences of the impact of the lack of NEK6 in cells include important alterations in mitochondrial fission/fusion equilibrium, the mitochondrial-related autophagic processes as well as endoplasmic reticulum–mitochondria contacts.

**Abstract:**

The family of Nek kinases has 11 human members that are conserved in their kinase domains but diverse in their regulatory domains. Functionally, they can be associated with diverse aspects of cell cycle regulation, from mitosis and primary cilia function to centrosome disjunction in the G2 phase and checkpoints of the DNA damage response. However, novel functional contexts have emerged in recent years, including regulatory roles of Neks 1, 4, 5, and 10 in mitochondrial metabolic and morphological homeostasis. We recently generated, by CRISPR-Cas9 technology, a DU-145 prostate cancer cell line, with an NEK6 gene knockout. Here, we focus on a detailed characterization of changes in this cell line, in mitochondrial respiration function and morphology. DU-145 NEK6 knockout cells exhibited reduced mitochondrial respiration and a fragmented phenotype in electron microscopy, with reduced mitochondrial cristae numbers. Alterations in mitochondrial architecture and respiration were correlated with increased expression of anaerobic glycolytic proteins (HK2, PFKP, and LDHA) and decreased expression of PDH, an enzyme of aerobic glycolysis. Molecular analysis by Western blot revealed decreased levels of mitochondrial mass and biogenesis protein markers (TOM20, TFAM), without alterations in other markers such as VDAC1/3 or mtDNA copy number in the NEK6 knockout cells. Furthermore, the regulators of mitochondrial fusion/fission are altered in the knockout cells (decrease in the Long-OPA1:Short-OPA1 ratio and DRP1 total level), which is associated with an increase in endoplasmic reticulum–mitochondria contact at ≤20 nm observed in transmission electron microscopy (TEM) image analysis. Using analysis of TEM micrographs, we found an increase in the autophagic structures (autophagosome, amphisome, and autolysosome), with mitochondria as cargo in some structures, which was correlated with a decrease in LC3A/B and an increase in the BECLIN1 total level, and with an increase in acidic vesicles approximation, suggesting that reduction in TOM20 and TFAM without alterations in VDAC1/3 and mtDNA copy number might be related to mitochondrial degradation through autophagy. Together, our data suggest a new role for NEK6 in regulating mitochondrial homeostasis, where its loss alters mitochondrial morphology and respiration, and could be associated with an increase in the degradation of the dysfunctional mitochondria through autophagy.

## 1. Introduction

The family of Nek kinases (never in mitosis gene A-related kinases) comprises 11 members primarily characterized as regulators of primary cilia function (NEK1, 8), mitosis (NEK6, 7, 9), centrosome disjunction (NEK2) and more recently of the DNA damage response in a wider sense (NEK1, 4, 5, 8, 10 and 11). Accumulating evidence, including studies from our group, has identified a new functional axis for Neks in the regulation of mitochondrial functions and structure (NEK1, 4, 5, and 10) [1].

NEK1 was the first family member to be associated with mitochondrial functions [2,3]. NEK1 was found to interact with VDAC1 (voltage-dependent anion channel 1) and to prevent mitochondrial-mediated apoptosis through phosphorylation of VDAC1 at Ser 193, on the outside of the mitochondrial membrane. Overexpression of a Ser193Ala mutant of VDAC1 induces cell death, whereas a Ser193Glu phosphomimetic mutant of VDAC1 is able to mediate cell survival transiently after exposure to DNA-damaging reagents. Also, the same research group found that NEK1-mediated VDAC1 phosphorylation maintains it in a closed configuration, preventing Cytochrome C efflux and cell death [3].

The second member of the family found to be involved in mitochondrial functions was NEK5 [4]. NEK5 was found to interact with several mitochondrial proteins, including COX11, MTX-2 and BCLAF1, as well as TFAM, LONP1, C2ORF47, MFN2, TFB2M and RAB32 [5]. When its expression was silenced or a kinase-inactive mutant of NEK5 was expressed in HEK293T cells, an increase in ROS formation was observed. Silencing NEK5 expression also caused an increase in mitochondrial basal respiration rates and apoptosis.

NEK10 interacts with mitochondrial proteins, including ATAD3A/B, ATP5J, GLUD1, and APEX1 [6]. NEK10 knockout in HAP1 cells causes mitochondrial fragmentation, impairment of respiration, and increased mtDNA damage [6]. In addition, NEK4 was found to interact with several mitochondrial proteins, including TRAP1, AIFM1, and ANT3 [7]. NEK4 depletion reduced mitochondrial respiration and mitochondrial elongation, whereas its overexpression was associated with increased respiration and elevated mitochondrial membrane potential [7]. Key regulators of mitochondrial fusion and fission are deregulated by NEK4 silencing or overexpression, including DRP1, OPA1, and MFN1/2.

Several other cell cycle-regulating kinases, such as CDKs and Aurora kinases, have been described to localize to mitochondria and regulate metabolic aspects of mitochondrial function. CDK1, for example, was reported to locate in the mitochondria and boosts ATP production via complex I, providing nucleotides for the mitochondrial DNA repair process of mtDNA [8]. Inhibition of CDK4/6 increases mitochondrial metabolism through enhanced mitochondrial respiration capacity [9]. Aurora kinase A was shown to regulate mitochondrial fusion and ATP production, mediated through CDK1 [10].

NEK6 is a serine/threonine protein kinase of 313 amino acids in length and functionally participates in mitotic spindle formation, cytokinesis, and G2/M transition checkpoints [1,11]. NEK6 is closely related but functionally independent of NEK7, and both are activated by NEK9, which exerts a central role in the regulation of the mitotic spindle. Other functions of NEK6 have been associated with signaling in general and with DDR, through CHK1/2 checkpoint signaling [12]. Protein interactomics have also pointed out several mitochondrial proteins as interactors of NEK6, suggesting that it would be yet another member of the NEK family associated with mitochondrial homeostasis regulation [13]. These include the proteins PRDX3 (a peroxiredoxin), DNAJC-19 (an HSP40 homolog at the mitochondrial membrane), and mitochondrial ribosome proteins L28 and L19.

Several cancer types and neurodegenerative diseases are related to NEK6 overexpression [14,15]. NEK6 is implicated in prostate cancer progression and development of castration-resistant prostate cancer (CRPC) [16], which mediates androgen-independent tumor growth. Interestingly, the pathophysiology, progression, and therapeutic resistance of CRPC rely on changes in mitochondrial functions [17,18]. Here, we characterized NEK6 knockout DU-145 cells regarding mitochondrial function and morphology. We demonstrated that NEK6 is involved in regulating mitochondrial function. We found that NEK6 knockout results in impaired mitochondrial respiration, which is associated with a mitochondrial fragmentation phenotype. Moreover, we observed an increase in endoplasmic reticulum–mitochondria contacts at ≤20 nm, an increase in autophagic structures, such as autophagosomes, amphisomes, autolysosomes and approximation between acidic vesicles, that were correlated with altered expression levels of proteins related to mitochondrial biogenesis, dynamics and autophagy, suggesting that mitochondrial respiration impairment and a fragmented phenotype induced by NEK6 knockout could be associated with increased mitochondrial fission and autophagy. This adds yet another Nek family member to the growing list of NEKs associated with mitochondrial functions and could represent a new axis to be explored in CRPC treatments.

## 2. Materials and Methods

### 2.1. Cell Culture

DU-145 wild-type and NEK6 knockout cell lines were maintained in RPMI 1640 (Thermo Scientific, #11875093, Waltham, MA, USA) medium supplemented with 10% fetal bovine serum (FBS, Thermo Fisher Scientific, Waltham, MA, USA, #12657029) and maintained at 37 °C in an incubator with a humidified atmosphere and 5% CO_2_. NEK6 knockout cells were used for experiments between passages 3 and 8 after confirmation of the knockout.

### 2.2. NEK6 Knockout Generation Using CRISPR-Cas9 System

The protocol was carried out as described by Ran and colleagues (2013) [19]. Two sgRNA sequences were designed using CRISPOR to knock out NEK6 in DU-145 cells. We chose guides with high specificity and efficiency scores; the criteria were determined by Doench and co-workers [20]. The sgRNA targets exon 3 of the NEK6 gene, which was called 83 forward sgRNA (sg83) with the sequence 5′-AGGCCGAGGACAGTTCAGCG-3′, and exon 7 of the NEK6 gene, named 56 reverse sgRNA (sg56), with the following sequence: 5′-TGAAGAAGCGGCCCAGACCA-3′. Oligos were cloned into the PX459 vector (SpCas9(BB)-2A-Puro V2.0, Addgene, Watertown, MA, USA, #62988); transfected in DU-145 cells using 2500 ng DNA, Lipofectamine™ (Thermo Scientific, #18324012, Waltham, MA, USA), and Plus™ reagent (Thermo Scientific, #11514015, Waltham, MA, USA); and cultured with 1 μg/mL puromycin for 72 h to select positively transfected cells. Positively transfected cells were seeded into 96-well plates at 0.5 cells per well. The resulting monoclonal cultures were screened by Western blot for the loss of NEK6 expression using an NEK6 antibody (Santa Cruz, Dallas, TX, USA, sc-50752). To evaluate the indels generated by the CRISPR-Cas9 system, the targeted genomic region for NEK6 was amplified by PCR from genomic DNA and sequenced. The indels were characterized by cloning the amplicons into the pGEM-T vector (Promega, Madison, WI, USA, #A3600), followed by Sanger-type sequencing and analysis of up to four different chromatograms.

### 2.3. Oxygen Consumption

Cellular oxygen consumption was measured using a high-resolution oxygraph chamber (OROBOROS Oxygraph-2 K, Oroboros Instruments GmbH, Innsbruck, Austria). DU-145 wild-type and NEK6 knockout cell lines were trypsinized, resuspended in RPMI 1640 (Thermo Fisher Scientific, Waltham, MA, USA, #11875093) medium with 10% FBS (Thermo Fisher Scientific, Waltham, MA, USA, #12657029), and 10^6^ cells were incubated in a closed chamber containing 2 mL of the same medium under agitation. The baseline oxygen consumption (oxygen flow) was monitored, and to analyze the consumption coupled to oxidative phosphorylation (ATP-linked respiration), maximum mitochondrial respiration, and non-mitochondrial oxygen consumption, 1 μM oligomycin, 1  μM Carbonyl cyanide m-chlorophenyl hydrazone (CCCP), and 1 μM rotenone were added, respectively. The ATP-linked respiration was measured by the difference in oxygen consumption rate (OCR) baseline and after addition of oligomycin. The maximal oxygen consumption capacity was measured after the addition of CCCP (a chemical mitochondrial protonophore, uncoupling the oxygen consumption) and the spare capacity was accessed through the difference in basal oxygen consumption and maximal oxygen consumption. Proton leakage was measured through the difference in the OCR after administration of rotenone (non-mitochondrial oxygen consumption) from OCR after oligomycin administration. Data were acquired using DatLab 4 software (OROBOROS Instruments, Innsbruck, Austria), and were statistically analyzed using the Shapiro–Wilk test, with the One-way ANOVA test followed by Tukey’s multiple comparison test.

### 2.4. Transmission Electron Microscopy (TEM): Monolayer in Epon Resin

DU-145 wild-type and NEK6 knockout cell lines were plated in 6-well plates under a glass coverslip until reaching 80% of confluence and fixed with 2.5% glutaraldehyde in 0.1 M sodium cacodylate and 3 mM CaCl_2_ buffer at room temperature for 5 min, followed by a 1 h incubation on ice. The samples were washed three times in 0.1 M sodium cacodylate and 3 mM CaCl_2_ buffer, and incubated for 30 min in 0.1 M sodium cacodylate buffer containing 1% osmium tetroxide and 0.8% potassium ferrocyanide, and in bloc stained with 1% uranyl acetate overnight at 4 °C. The cells were dehydrated with ethanol at 4 °C and infiltrated with Epon resin. After four changes of resin solution, a fifth change was carried out and the resin was immediately placed in a vacuum oven at 60 °C for 72 h. Ultrathin sections were cut with a Leica Ultracut microtome, stained with 2% uranyl acetate and Reynolds lead citrate, and examined in a Tecnai G2 Spirit BioTWIN (FEI Company, Hillsboro, OR, USA) transmission electron microscope at an accelerating voltage of 60 kV. TEM was carried out at the Electron Microscopy Laboratory of the Institute of Biology at the University of Campinas. The morphometric analyses of the mitochondria, endoplasmic reticulum (ER)–mitochondria contact site distance, and autophagic structures were carried out in 7 cells from each group (DU-145 wild-type and knockout NEK6) with software ImageJ (v1.54s), using magnifications of 1900× from each cell with magnifications of 1900×, 2900×, 4800×, 9300×, 11,000×, and 13,000× from several regions of the same cell. Notwithstanding the sample size analyzed through TEM images, it was possible to statistically differentiate the phenotype observed after NEK6 knockout in the DU-145 cell line.

We analyzed mitochondrial morphology and ER–mitochondria contact sites (ERMCSs) as described by Lam et al. (2021) [21] with modifications. Mitochondrial morphology analysis was carried out on mitochondria with well-defined outer and inner mitochondrial membranes with at least one crista. We observed approximately 200 mitochondrial sections from 7 cells (ranging from 15 to 40 mitochondrial sections from each cell analyzed). The ERMCSs were analyzed using several distances measured in parallel during contact between the endoplasmic reticulum membrane and the mitochondrial outer membrane. We looked for a good definition of ER and mitochondria membranes, where we analyzed 100 contacts between ER and mitochondria from 7 cells, drawing several distances in parallel in each contact (ranging from 10 to 100 distances from 5 to 20 ERMCSs of each cell analyzed, totaling approximately 3000 distances in the analysis), and the distances above 60 nm were removed.

The autophagic structural analysis was carried out through the identification of phagophores, autophagosomes, amphisomes, and autolysosomes [22,23,24]. Briefly, the phagophores were identified as a bilayer in a cup shape (C-shape), not sealed, may have cisterns near the opening and engulfing some cytoplasmic content. Omegasomes (structures formed between endoplasmic reticulum and phagophore membranes) were identified and counted as phagophores. The autophagosome and amphisome were identified as a sealed vacuole with a lipid bilayer and cargos (ER, mitochondria, ribosomes, and membranes) without electron-dense structures inside the vacuoles. Amphisomes were discriminated from autophagosomes through the cytoplasmic content observed inside the autophagosomes and not in amphisomes, such as ribosomes. Also, amphisomes were discriminated from endosomes through their cargos, where organelles and membranes were observed inside vacuoles, not in endosomes. Early and late endosomes (sealed bilayers without cargos) were not counted. The autolysosomes are formed by fusion of autophagosome or amphisome with lysosomes, and were identified as a sealed bilayer with cargos (ER, mitochondria, ribosomes, and membranes) with electron-dense structures inside vacuoles due to acidification and degradation of cargos. Amphisomes can also acidify the vacuole, degrading the cargos and increasing electron-dense structures inside the vacuoles. We counted amphisomes with electron-dense structures such as autolysosomes. The phagophore, autophagosome, amphisome, and autolysosome numbers were calculated through the cytoplasm area (μm^2^) by subtraction of the nucleus area from the cellular area from TEM images.

### 2.5. Whole-Cell Lysate

DU-145 wild-type and NEK6 knockout cell lines were trypsinized, washed twice in cold PBS, resuspended in lysis buffer (50 mM Tris pH 7.4, 150 mM NaCl, 0.1% NP-40, supplemented with protease and phosphatase inhibitors), sonicated in ice-water bath (5 cycles of 2 s on/10 s off at 20% Wmax), and centrifuged for 10 min 10,000× *g* at 4 °C. Protein concentration was determined by Bradford assay.

### 2.6. Immunoblot

Thirty micrograms of whole-cell lysates were resolved by SDS-PAGE, transferred to nitrocellulose membranes (Amersham, GE Healthcare Life Sciences, Pittsburgh, PA, USA) and incubated overnight at 4 °C with the following primary antibodies: NEK6, HK1, HK2, PFKP, PKM1/2, LDHA, PDH, VDAC1/3, OPA-1, MFN1, MFN2, DRP1, TOM20, TFAM, LC3A/B, BECLIN1, TUBB, and GAPDH. The secondary antibodies were incubated for 2 h at room temperature, and protein detection was carried out using the enhanced chemiluminescence ECL Western Blotting System (Amersham, GE Healthcare Life Sciences, Pittsburgh, PA, USA) in Chemidoc.

### 2.7. Relative Mitochondrial DNA Copy Number

Total cellular DNA was isolated using the DNeasy Mini Kit (Qiagen, Hilden, Germany), and DNA concentrations were determined spectrophotometrically with Nanodrop. Equal amounts of total DNA were assayed by quantitative PCR (qPCR) with qPCRBIO SyGreen Mix separate ROX (#PB20, 14-05, PCR Biosystems, London, UK). All reactions were performed in a CFX384 touch real-time PSR detection system (Biorad, Hercules, CA, USA). The comparative Ct method was applied to quantify mitochondrial DNA copy number by comparing the amplification of ND1 (mitochondrial gene) to B2M (nuclear gene). Primer pair sequences are as follows: ND1-F 5′-ACTACGCAAAGGCCCCAACG-3′; ND1-R 5′-GAGCTAAGGTCGGGGCGGTG-3′; B2M-F 5′-TGCTGTCTCCATGTTTGATGTATCT-3′; B2M-R 5’-TCTCTGCTCCCCACCTCTAAG T-3’.

### 2.8. Fluorescence of Acridine Orange

DU-145 wild-type and NEK6 knockout cells were seeded in 96-well imaging plates and incubated for 24 h under standard culture conditions (37 °C, 5% CO_2_) to allow cell adhesion and stabilization. Based on preliminary titration assays, 20 µM of acridine orange was selected as the optimal concentration for DU-145 WT and NEK6 KO cells. Cells were incubated at 37 °C and 5% CO_2_ for 10 min with 20 µM of acridine orange. The staining solution was subsequently aspirated and replaced with 100 µL of medium containing Hoechst 33342 (1 µg/mL; Thermo Fisher Scientific, Waltham, MA, USA), followed by a 20 min incubation for nuclear labeling. Live-cell imaging was carried out using a Biotek Cytation™ 5 Hybrid Multi-Detection Reader (Agilent, Santa Clara, CA, USA, software: Gen5, v3.12) equipped with environmental control to maintain 37 °C and 5% CO_2_, conditions previously optimized to preserve cell viability and morphology during acquisition. Images were acquired using a 20× objective with the following fluorescence settings: GFP (469/525 nm), PI (531/647 nm), and DAPI (377/447 nm). A minimum of nine fields of view per well were collected. Autofocus was performed preferentially using the DAPI channel to ensure robust and reproducible focal plane detection; the resulting focal parameters were then applied across all channels to maintain spatial alignment. Alternatively, autofocus was carried out using the GFP channel and propagated to the remaining channels. The images were analyzed using ImageJ, and the Nearest Neighbor Analysis of spots was carried out according to Ecker et al. [25], with modifications, where we used the mean of the nearest neighbor distance of all spots of each cell.

### 2.9. Statistical Analysis

Statistical analysis was carried out using RStudio (v2025.9.1.401). The normality of the data was assessed using the Shapiro–Wilk test; normally distributed data were compared using the unpaired *t*-test, and non-normally distributed data were analyzed using the Mann–Whitney test. A One-way ANOVA followed by Tukey’s multiple comparison test was carried out in cellular respiration analysis. Data are shown as mean ± SD, or median ± IQR. The differences with *p* < 0.05 were considered statistically significant.

## 3. Results

### 3.1. NEK6 Knockout Impairs Mitochondrial Respiration and Alters Protein Levels of the Anaerobic and Aerobic Glycolytic Pathway

**Figure 1:**
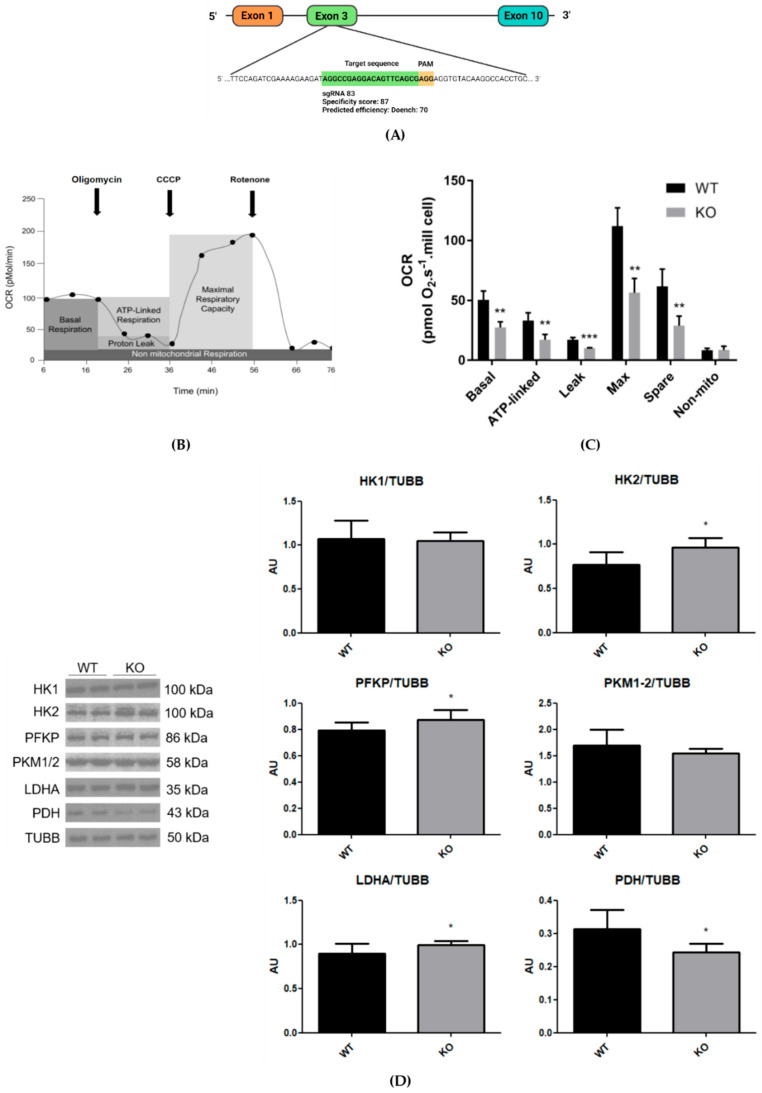
NEK6 knockout was performed in DU-145 cells using the CRISPR-Cas9 technique, and mitochondrial respiration and glycolytic protein levels were affected. (**A**) Target sequence of NEK6 (green) followed by the PAM sequence (orange). (**B**) Experimental scheme of oxygen consumption rate (OCR) by Oxygraph-2K OROBOROS, adapted from Rose et al. (2014) [27]. (**C**) The graph shows the comparison of oxygen consumption (pmol O_2_·s^−1^·10^6^ cells) of ATP-linked respiration, proton leak, maximal respiratory capacity, spare respiratory capacity, and non-mitochondrial oxygen consumption. The analyses were performed in 5 independent experiments using 10^6^ cells in an OROBOROS chamber. Statistical analysis was conducted using the Shapiro–Wilk test for normality analysis with One-way ANOVA test followed by Tukey’s multiple comparison test (** = *p* < 0.05 and *** = *p* < 0.005). (**D**) Western blot analysis of HK1, HK2, PFKP, PKM1/2, LDHA, and PDH using TUBB as loading control from the total lysate of wild-type and NEK6 knockout DU-145 cell line. Densitometric analysis of the bands was performed using ImageJ. Data are shown as mean ± SD. Statistical analysis was conducted using the Shapiro–Wilk test for normality and an unpaired *t*-test for analysis of the two groups. * = *p* < 0.05. AU = arbitrary unit.

Since NEK6 has important functions in the development of CRPC, the DU-145 cell line was chosen, representing one of the CRPC models in the literature, due to very low androgen receptor levels [26]. Since gene knockout is an established approach to analyze and validate gene function, we generated an NEK6 gene knockout in DU-145 cells, as described previously [11], which no longer expresses any NEK6 protein (Figure 1A).

The OROBOROS oxygen consumption assay represents a precise measurement of real-time oxygen consumption in cells. To investigate the effects of NEK6 deletion on oxygen consumption rate (OCR), we performed the Oxygraph-2K OROBOROS experiment in DU-145 cells and the NEK6 knockout version, in the presence of inhibitors according to the oriented methodology [27]. Figure 1B shows typical OCR measurements using OROBOROS, and, with the exception of non-mitochondrial oxygen consumption, all other measured parameters were significantly decreased in NEK6 knockout cells compared with control cells (Figure 1C).

The basal oxygen consumption and ATP-linked respiration are decreased in NEK6 knockout cells (Figure 1C). The maximal oxygen consumption capacity was significantly lower in NEK6 knockout cells than in control cells (Figure 1C), indicating therefore a lower maximal mitochondrial electron transfer capacity in cells with NEK6 knockout. The spare capacity was markedly reduced in NEK6 knockout cells compared to control, indicating that cells lacking NEK6 are more prone to the exhaustion of respiratory reserve capacity due to a lower capacity of oxygen consumption adjustment to cell metabolic demands.

The addition of rotenone and antimycin A inhibits complex I and II of the ETC, increasing NADH and FADH_2_ levels and reducing the NAD^+^/NADH and FAD/FADH_2_ ratio, which in turn reduces the mitochondrial membrane potential and the oxidoreduction reactions of the Krebs cycle, thereby inhibiting the mitochondrial respiration [28]. The use of rotenone alone may not be sufficient to inhibit the oxygen consumption because complex II can feed the ETC in the presence of rotenone in intact cells. However, after the addition of rotenone only, the non-mitochondrial oxygen consumption (non-mito) did not show differences between NEK6 knockout and control cells (Figure 1C), indicating limited residual ETC flux in the absence of antimycin A.

Following the analysis of OCR, the proton leak (leakage) indicates diffusion of protons in mitochondrial intermembrane space to the mitochondrial matrix through channels, like ANT1 and UCP1, that uncouple the oxygen consumption from ATP production by mitochondria [29]. The proton leakage was not different between NEK6 knockout and control cells (Figure 1C) indicating that OCR referred to ATP is not associated with mitochondrial uncoupling. In summary, these results indicate that NEK6 can functionally regulate OXPHOS. The data also suggest that NEK6 depletion can alter mitochondrial function, either directly or indirectly through its interactants, via protein–protein interactions or by disrupting NEK6-dependent phosphorylation.

Alterations in glucose catabolism can accompany alterations in mitochondrial respiration. The glycolytic pathway is divided into anaerobic and aerobic glycolysis, where anaerobic glycolysis phosphorylates and breaks down a glucose molecule into two molecules of pyruvate in the cytosol, which are reduced to lactate by LDHA [30]. In aerobic glycolysis, the pyruvate is decarboxylated to acetyl-CoA by PDH in mitochondria to proceed with the Krebs cycle and reduction in the coenzymes NAD^+^ and FAD to participate in OXPHOS [30]. We observe a slight but significant increase in the protein levels of HK2, PFKP, LDHA, and a reduction in PDH in the NEK6 knockout cells (Figure 1D). The alterations in HK2, PFKP, and LDHA protein levels can be a compensation in the anaerobic glycolysis pathway due to the reduction in mitochondrial respiration (Figure 1D); however, the glycolytic flux needs to be assessed because these enzymes are allosterically regulated, and the increase in glycolytic flux may not accompany their levels.

### 3.2. NEK6 Knockout Changes Mitochondrial Morphology

**Figure 2:**
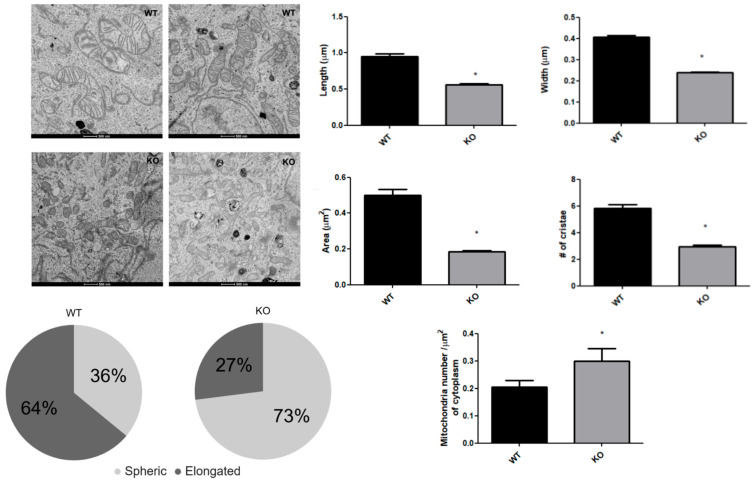
Mitochondrial morphology is affected by NEK6 knockout. Transmission electron microscopy images of DU-145 wild-type and NEK6 knockout cells were used at a magnification of 1900× to count mitochondrial number per cytoplasmic area of the cell, and magnifications of 4300×, 9300×, and 11,000× from several areas of the same cell were used to confirm the mitochondria counted. The mitochondria were identified by lipid bilayer (outer and inner mitochondrial membrane), and cristae were identified as invaginations formed by the bilayers inside the mitochondrial structure. Scale bar of 500 nm. Bar graphs showing the comparison of length, width, area, number of cristae of the mitochondria, and mitochondrial number per cytoplasmic area of the cell, and the pie chart shows the relative amount of mitochondria elongated compared to spherical, both for wild-type and NEK6 knockout. The morphometric quantifications were performed using the software ImageJ. We analyzed 1 cell section from 7 cells per group, including 200 mitochondria sections from 7 cells. Statistical analysis was conducted with normality analysis using the Shapiro–Wilk test and Mann–Whitney test. Data are shown as median ± IQR. * = *p* < 0.05.

The functional alterations in mitochondrial respiration and glycolytic enzymes in the NEK6 knockout cells, reported above, suggested that they may be accompanied by observable morphological changes in the mitochondria. Such a notion is further suggested by our previous observations that a knockout or knockdown of NEK1, 4, or 10 also resulted in both alteration in the mitochondrial respiration and morphology [6,7]. Since morphological and ultrastructural modifications were observed for NEK1, 4, 5 and 10 knockouts or knockdown cells, we used transmission electron microscopy images of DU-145 wild-type and NEK6 knockout cells to document morphological changes within the cells (Figure 2).

We found visible alterations in the overall and internal morphological features of the mitochondria in the NEK6 knockout cells. The average mitochondrial length and width were approximately 30% lower in the knockout cells (Figure 2). Also, the mitochondrial area was reduced by 60% and the average number of cristae by 50% in the NEK6 knockout cells (Figure 2). As a consequence of change in mitochondrial length, when dividing mitochondria into two classes (spherical vs. elongated shape, less than 0.6 μm in length, respectively), we found an inversion from a predominantly elongated (64%) phenotype in wild-type cells to a predominantly spherical phenotype (72.5%) in NEK6 knockout cells. This spherical phenotype of mitochondria was accompanied by an increase in the number of mitochondria sections per cytoplasm area, indicating that NEK6 knockout induces a mitochondrial fragmentation phenotype. Indeed, mitochondrial fragmentation is associated with a reduction in oxygen consumption [31]. Since the cristae numbers are correlated to the expansion of mitochondrial inner membrane, where the complexes of ETC are located [32], the reduction in cristae number observed after NEK6 knockout may be related to the reduction of all parameters in oxygen consumption (Figure 1C).

### 3.3. Alterations in Mitochondrial Morphology in NEK6 Knockout Cells Are Associated with Alterations of Mitochondrial Mass, Biogenesis, and Dynamic Protein Markers, and Alterations in Autophagy Protein Markers with an Increase in Autophagy Structures and Approximation Between Acidic Vesicles

After detecting both reduced mitochondrial respiration and morphological defects in the NEK6 knockout cells, to further characterize the molecular basis of this phenotype we assessed classical protein markers of mitochondrial biogenesis, dynamics (fission and fusion), and degradation (autophagy), since the alterations between these markers can correlate with alterations in these processes that show a straight connection with mitochondrial functions and morphological modifications.

Mitochondrial biogenesis is a process that increases mitochondrial size (mass) or number and mitochondrial DNA (mtDNA) transcription and replication, which are associated with an increase in mitochondrial metabolic functions [33]. The proteins, TOM20 and TFAM, are indicative markers of relative mitochondrial mass and mtDNA replication, transcription, and stability, respectively [34,35]. VDAC is the most abundant protein of the mitochondrial outer membrane related to the regulation of metabolic, morphological, and death properties of the mitochondria [36]. The mtDNA is duplicated during mitochondrial biogenesis, and TFAM has functions in mtDNA replication and transcription, being important to mitochondrial biogenesis [33]. Under such conditions, mitochondrial biogenesis increases protein levels of VDAC, TOM20, and TFAM [37].

**Figure 3:**
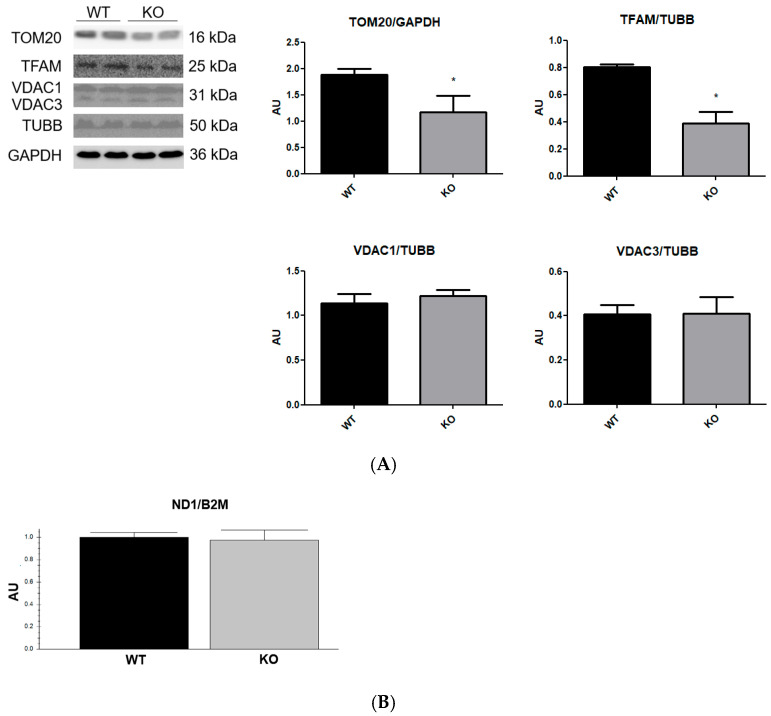
Analysis of proteins related to mitochondrial mass and biogenesis, and mtDNA copy number of NEK6 knockout in the DU-145 cell line. (**A**) Western blot analysis of TOM20, TFAM and VDAC1/3 using TUBB and GAPDH as loading controls from the total lysate of wild-type and NEK6 knockout DU-145 cell line. Densitometric analysis of the bands was performed using ImageJ. Data are shown as mean ± SD. Statistical analysis was conducted using the Shapiro–Wilk test, and normality analysis was conducted using the unpaired *t*-test. * = *p* < 0.05. AU = arbitrary unit. (**B**) qPCR of mitochondrial gene ND1 using nuclear gene B2M as loading control. Data are shown as mean ± SD. Statistical analysis was conducted using the Shapiro–Wilk test, and normality analysis was conducted using the unpaired *t*-test.

The content of TOM20 and TFAM was markedly reduced in NEK6 knockout cells (Figure 3A); on the other hand, total levels of proteins VDAC1/3 (Figure 3A) and mtDNA copy number (Figure 3B) were not altered with NEK6 knockout. Therefore, the reduction in TOM20 levels could be associated with the reduction in mitochondrial mass in NEK6 knockout cells; however, VDAC1 is also an indicator of mitochondrial mass and was not altered after NEK6 knockout. Also, the reduction in TFAM levels could be associated with the reduction in mitochondrial biogenesis; however, the mtDNA is replicated during mitochondrial biogenesis [37], and does not show alteration after NEK6 knockout. And therefore, the reduction in TOM20 also could not be associated with an alteration in mitochondrial biogenesis, since VDAC1 levels are also altered by mitochondrial biogenesis [37]. On the other hand, reductions in TOM20 without alterations in VDAC1 were observed in cancer cells after degradation of dysfunctional mitochondria by mitophagy [38].

For mitochondrial degradation to occur through mitophagy, changes in mitochondrial dynamics may be necessary, as fission events remove damaged components from the mitochondria for degradation via autophagy machinery.

The endoplasmic reticulum–mitochondrial (ER-Mito) contact sites (ERMCSs) are regions of endoplasmic reticulum membrane near the mitochondrial membrane, which are related to diverse aspects of mitochondrial metabolism, such as mtDNA maintenance and replication, phospholipid transport, calcium handling, as well as mitochondrial fission and fusion events [39,40]. The ERMCSs can be classified into 30 nm and 20 nm distances between the organelles, where distances of less than 20 nm were associated with mitochondrial fission and fusion events. ERMCSs with ≤20 nm are regulated by the interaction between VDAC1, IP3R, and GRP75 and between MFN1 and MFN2 [41]. The fusion of mitochondria is regulated by MFN1, MFN2, and OPA1, where interactions between MFNs are related to the fusion of the mitochondrial outer membrane, and OPA1 with mitochondrial inner membrane fusion [31]. During the process of mitochondrial fission, proteins in the outer membrane of mitochondria interact with proteins of the endoplasmic reticulum membrane that surround the mitochondria to constrict their membrane and reduce their diameter to fission. The DRP1 and actin filaments mediate the constriction force to fission of mitochondria through the formation of a ring complex of DRP1 around mitochondria, which localizes at the interface of ERMCSs [39,42].

**Figure 4:**
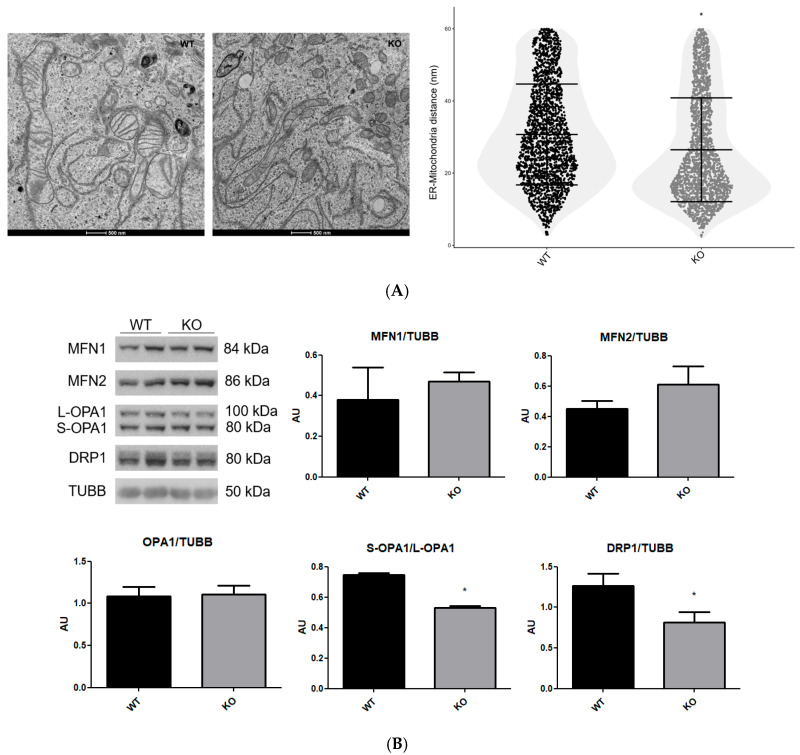
Mitochondrial morphologic and functional alterations of NEK6 knockout in the DU-145 cell line are associated with an increase in ERMCSs with ≤20 nm and alteration in mitochondrial dynamic protein levels. (**A**) Electron microscopy images of DU-145 wild-type and NEK6 knockout cells, at a magnification of 9300× and a scale bar of 500 nm. Graphs show the comparison of several distances measured in parallel throughout the ER-Mito interfaces using ImageJ. Approximately 3000 distances were analyzed in parallel from 100 ER-Mito contacts from 7 cells per group. Statistical analysis was conducted with normality analysis using the Shapiro–Wilk test and the Mann–Whitney test. Data are shown as median ± IQR. * = *p* < 0.05. (**B**) Western blot analysis of MFN1, MFN2, OPA1, and DRP1 using TUBB as loading control from the total lysate of wild-type and NEK6 knockout DU-145 cell line. Densitometric analysis of the bands was performed using ImageJ. Data are shown as mean ± SD. Statistical analysis was conducted using the Shapiro–Wilk test, and normality analysis was conducted using the unpaired *t*-test. * = *p* < 0.05. AU = arbitrary unit.

The transmission electron microscopy images can be used to access the ERMCSs distance, because the mitochondrial and reticulum structures and these distances can be accurately determined [21]. We found an increase in ERMCSs with ≤20 nm observed in electron microscopy images (Figure 4A), and an analysis of the total level of the proteins MFN1, MFN2 and DRP1 showed a reduction in DRP1 and an increase, but not statistically significant, in MFN2 (*p* = 0.06) without alterations in MFN1, with a reduced Long-OPA1:Short-OPA1 ratio in NEK6 knockout cells (Figure 4B).

Mitochondrial depolarization or permeability transition induces the cleavage of Long-OPA1 by OMA1 to form Short-OPA1 [43]. The Long-OPA1 has a function in the fusion of the mitochondrial inner membrane, while the Short-OPA1 has this function inhibited [44,45]. Therefore, the reduction in the ratio Long-OPA1:Short-OPA1 might be related to mitochondrial dysfunction in NEK6 knockout cells, suggesting that the mitochondrial matrix of the NEK6 knockout cells is less prone to mitochondrial fusion. And how MFN1/2 and VDAC1 function in the formation of ERMCSs with ≤20 nm, the increase in ERMCSs with ≤20 nm associated with maintenance of VDAC1 and MFN1 total levels and increasing trend in MFN2 suggest that the approximation of mitochondria to endoplasmic reticulum could be related to mitochondrial fragmentation. Also, the maintenance of mtDNA copy number is related to mitochondrial cristae number and TFAM levels [46,47], and the reduction in mitochondrial cristae number and TFAM levels was not accompanied by an mtDNA copy number reduction in NEK6 knockout. Interestingly, ERMCSs are related to maintenance and replication of mtDNA copy number [40], and the reduction in TFAM levels induces an increase in ERMCS approximation [47]; therefore, the ERMCS distances in the NEK6 knockout could be related to maintenance of mtDNA copy number.

Interestingly, the DRP1 levels generally suffer an increase after mitochondrial dysfunction [48,49]. On the other hand, it was identified that reduction in DRP1 levels through proteolytic degradation can be induced by overexpression of PARKIN [50], a protein that seems to be induced upon mitochondrial dysfunction, during mitophagy. Therefore, the DRP1 reduction and MFN2 increase might be a cellular compensatory mechanism to reduce the excessive mitochondrial fragmentation. Therefore, the mechanisms underlying mitochondrial dynamics in NEK6 knockout cells need to be elucidated in greater depth.

**Figure 5:**
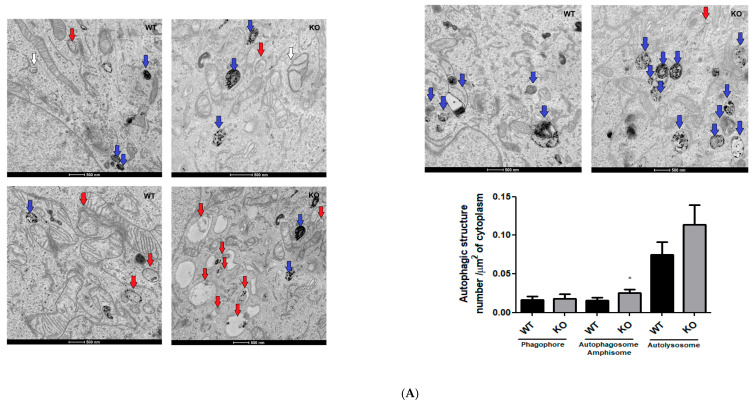

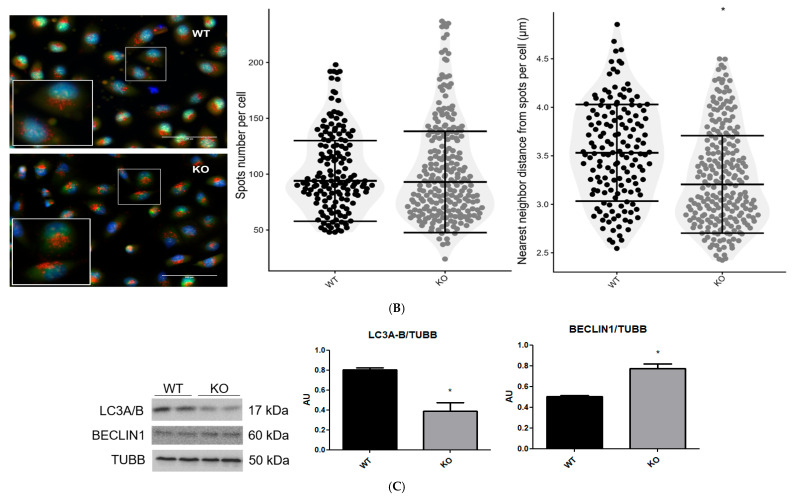
Mitochondrial morphologic and functional alterations of NEK6 knockout in the DU-145 cell line are associated with an increase in autophagic structures, acidic vesicles approximation, and alteration in autophagy protein levels. (**A**) Electron microscopy images of DU-145 wild-type and NEK6 knockout cells at a magnification of 9300× and a scale bar of 500 nm. Graphs show the comparison of phagophores (white arrow), autophagosomes/amphisomes (red arrow), and autolysosomes (blue arrow) per cytoplasmic area of the cell. One cell section from 7 cells per group was analyzed. Statistical analysis was conducted using the Shapiro–Wilk and Mann–Whitney tests. Data are shown as median ± IQR. * = *p* < 0.05. (**B**) Confocal images of acridine orange stain fluorescence demonstrating the acidic vesicles’ (red spots) cellular distribution. The spots’ number and nearest neighbor distance mean were processed in ImageJ, as by Ecker et al. [25], with modifications. Data are shown as median ± IQR. Statistical analysis was conducted using the Mann–Whitney test. * = *p* < 0.05. (**C**) Western blot analysis of LC3A/B and BECLIN1 using TUBB as loading control from the total lysate of wild-type and NEK6 knockout DU-145 cell line. Densitometric analysis of the bands was performed using ImageJ. Data are shown as mean ± SD. Statistical analysis was conducted using the unpaired *t*-test. * = *p* < 0.05. AU = arbitrary unit.

So, we looked for alterations in autophagy structures through analysis of electron microscopy images, levels of protein markers of autophagy, and acidic vesicles staining (Figure 5). We analyzed the numbers of phagophores, autophagosomes, amphisomes, and autolysosomes through electron microscopy [22]. The identification of autophagy structures by electron microscopy is the gold standard for this analysis because it is possible to identify the membrane structure and cargo of autophagosomes, amphisomes, and autolysosomes [22,23]. During the autophagic process, phagophores engulf cytoplasmic content, forming autophagosomes that can subsequently fuse with lysosomes, forming autolysosomes where the cargos are degraded by acidic hydrolases. Autophagosomes can be fused with endosomes, forming an amphisome, and subsequent acidification or fusion with lysosome (forming an autolysosome) is how mammalian cells degrade the autophagosome cargos [24].

In our analysis of autophagic structures (Figure 5A), we counted autophagosome and amphisome structures together because cytoplasmic contents were observed inside a lipid bilayer vacuole without an acidic lumen (electron-dense). We found a significant increase in autophagosome/amphisome and an increase, but not statistically significant, in the number of autolysosomes per area of cytoplasm (*p* = 0.06) in NEK6 knockout cells compared to control cells (Figure 5A), making it possible to identify mitochondria as cargo in some of these structures. The phagophore number per area of cytoplasm was not altered between NEK6 knockout and control cells (Figure 4A).

It has already been identified that acidic vesicles undergo retrograde movements within the cell cytoplasm during autophagy, and these movements increase the approximation between vesicles to enable the fusion of membranes and organelles, such as the lysosome and autophagosome [51]. Acridine orange is a membrane-permeable fluorescent dye that becomes protonated and accumulates in intracellular acidic vesicles (such as the lysosomes and autolysosomes) due to the low pH, emitting red fluorescence at high concentrations [52,53]. We used the red fluorescence of acridine orange to analyze the quantity of acid vesicles and the distance between them. We did not identify the statistical difference in the vesicle quantity between DU-145 NEK6 knockout cells compared to control cells (Figure 5B). Still, the mean of the nearest distance between the acid vesicles showed a reduction, indicating that the acid vesicles were closer together (Figure 5B).

Also, we found BECLIN1 protein levels significantly increased, while the LC3A/B total level significantly decreased in NEK6 knockout cells (Figure 5C). BECLIN1 is a protein of initiation of the autophagy pathway, which is characterized by the formation of phagophores and phagosomes with lipidation of LC3I to LC3II [22]. LC3 is an adapter protein with 3 isoforms (LC3A, LC3B, and LC3C) that are lipidated to LC3II at phagophores and phagosomes, which are related to the identification of cargos that are destined for degradation, such as mitochondria [24]. Interestingly, Wang et al. (2024), in a cellular model of ischemia–reperfusion injury, found that knockdown of the NEK6 levels with siRNA induces activation of the autophagy pathway [54]. The authors found that NEK6 knockdown increases BECLIN1 levels and phosphorylation of ULK1 at ser317 and AMPK at thr172, with an increase in LC3II and a reduction in LC3I associated with a decrease in phosphorylation of AKT at ser473 and mTOR at ser2448 [40]. Also, in the same work, it was identified that NEK6 overexpression had the opposite effect on autophagy pathway activation and was associated with an increase in autophagosome and a reduction in autolysosome formation in this cellular model, indicating a lysosomal dysfunction [54].

Therefore, while transmission electron microscopy has inherent limitations for identifying autophagic structures, the convergence of ultrastructural, biochemical, and molecular data provides robust support for autophagy-related alterations. The increased abundance of autophagosomes/amphisomes and autolysosomes, closer apposition of acidic vesicles, elevated BECLIN1 levels, and reduced LC3A/B expression suggest that the mitochondrial fission phenotype and reduced mitochondrial mass markers in NEK6 knockout cells are associated with autophagy activation and altered autophagic flux.

Moreover, as the disposal of dysfunctional mitochondria by mitophagy in cancer cells can reduce TOM20 without alterations in VDAC1 [38], this NEK6 knockouts could be related to an increase in mitochondrial degradation.

## 4. Discussion

The 11 members of the human Nek kinase family have their canonical functions attributed to mitosis, primary cilia function, centrosome disjunction and the DNA damage response. However, many recent studies suggested novel functional contexts, such as the regulation of apoptosis, and so far, the mRNA splicing regulation by two NEKs (NEK2 and NEK4). So far, at least 4 NEKs have been implicated in the regulation of mitochondrial homeostasis: NEK1, 4, 5, and 10 [1].

We demonstrated that NEK6 regulates mitochondrial respiration and morphology in DU-145 cells. The NEK6 knockout reduced the oxygen consumption rate and induced a mitochondrial fragmentation phenotype. The mitochondria show a more spherical shape, with reduced length, width, area, and cristae number after NEK6 knockout. The increase in the number of mitochondria per area of cytoplasm in NEK6 knockout cells suggests an increase in mitochondrial fragmentation. We identified alterations in some mitochondrial biogenesis and mass protein markers, such as TFAM and TOM20, but other mitochondrial proteins (VDAC1/3) and mtDNA copy number were not altered, and the association with the increase in autophagic structures (such as autophagosome, amphisome, and autolysosome) with approximation between acid vesicles, with the increase in BECLIN1 protein levels and the decrease in the LC3A/B protein level suggests that the reduction in TFAM and TOM20 could be related with the increase in mitochondrial degradation via autophagy in NEK6 knockout cells.

Our group identified that NEK6 knockout in DU-145 cells reduces mitochondrial membrane potential using TMRE fluorescence, which is associated with a reduction in cell viability in an analysis that uses MTT, which is reduced to formazan in the mitochondria [11]. In the same work, alterations in total ROS, antioxidant enzymes, DNA damage levels, and apoptosis in DU-145 cell knockout to NEK6 [11] were identified. Therefore, the fragmented phenotype with a reduction in mitochondrial respiration parameters, mitochondrial mass markers and cristae number, shown here, together with the finding, of a reduced mitochondrial membrane potential in DU-145 cells, with knockout for NEK6 [11], suggest that NEK6 is important in mitochondrial homeostasis, and its deletion increases mitochondrial fragmentation and degradation.

The depletion of mitochondrial interactors of NEK6 [13], such as PRDX3 and mitochondrial ribosome proteins L28 (MRPL28), also induces a decrease in mitochondrial respiration [55,56], while DNAJC-19 can show the opposite effect [57]. MRPL28 knockdown in SU86 cells decreased COX-III and COX-IV protein levels and reduced oxygen consumption, but no alteration in mitochondrial membrane potential or mass was observed [56].

PRDX3 activity supports mitochondrial fusion to preserve its function and energy production, which reduces oxygen consumption, ATP production and mtDNA, associated with an increase in ROS formation and reductions in MFN1 and MFN2 in skeletal muscle cells [58]. Interestingly, our group found that NEK6 knockout in DU-145 reduces the levels of antioxidant proteins, such as PRDX3 and SOD1/2, and increases ROS formation, DNA damage response protein levels, such as γH2AX and ATM phosphorylated at ser1981, and apoptosis [11]. This suggests that the increase in total ROS and DNA damage response signaling in DU-145 can be related to mitochondrial alterations induced by NEK6 knockout. However, NEK6 depletion increases SAC signaling through fragile spindle formation [59]; therefore, both alterations (mitochondrial and spindle formation) can contribute to an increase in DNA damage response.

We identified reductions in the Long-OPA1:Short-OPA1 ratio that could be related to mitochondrial cristae loss in NEK6 knockout cells, denoting intramitochondrial alterations. Interestingly, DNAJC-19 depletion is related to the reduction in mitochondrial cristae and protein levels of Long-OPA1, but does not alter mitochondrial membrane potential in HEK293T [60]. On the other hand, also was observed that loss of function in DNAJC-19 reduces mitochondrial area and the number of mitochondrial cristae and induces mitochondrial fragmentation, but this is accompanied by an increase in mitochondrial membrane potential and respiration in cardiomyocyte-derived iPSCs [57]. The precise molecular mechanisms mediated by NEK6 to promote and maintain mitochondrial homeostasis in DU-145 cells, and whether the same alteration can be observed in other cancer cell lines, remain to be fully elucidated. However, the alterations observed in NEK6 knockout cells may be due to the loss of interaction and regulation at least in mitochondrial interactors of NEK6, such as PRDX3, MRPL28, and DNAJC-19.

**Figure 6:**
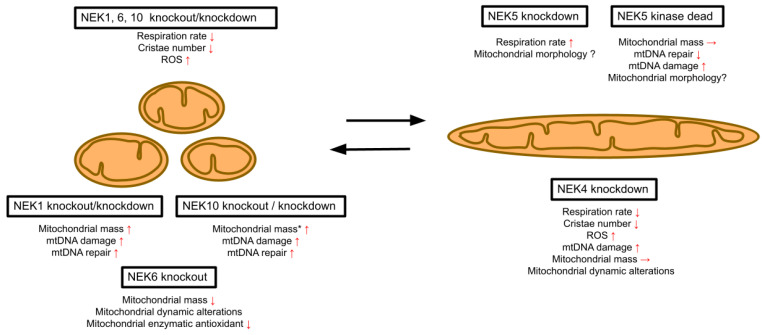
NEK1, 4, 5, 6, and 10 are involved in regulating mitochondrial homeostasis. Knockout or knockdown of NEK1, 4, 6, and 10 causes mitochondrial fragmentation, decreased respiration, and other phenotypes related to mtDNA damage, mitochondrial dynamics, DNA repair, apoptosis, ROS, and morphological phenotypes. NEK5 knockdown, on the contrary, causes an increased respiration rate. * = effect of NEK10 knockout. The red arrows indicate an increase, decrease, or no change in the observed parameter.

Interestingly, NEK6 signaling plays a central role in mediating castration-resistant prostate cancer, and the mitochondrial function is an important factor in the progression and therapeutic resistance of CRPC [17,18]. Therefore, elucidating the mechanisms underlying NEK6 signaling and the regulation of mitochondrial respiration and morphology can be important in the discovery of new targets in CRPC treatment. Also, future studies can explore mitochondrial and autophagic drugs in association with NEK6 inhibitors to give new insights into CRPC treatment approaches. Our findings position NEK6 within the established mitochondrial phenotype previously observed for most NEK family members, specifically NEK1, 4, 10, and partially NEK5 (Figure 6). Decreased respiration was also observed for the knockdown of NEK4 and NEK10 (Figure 6). Decreases in both mitochondrial mass and mitochondrial length were also observed in depletion experiments for NEK1 and NEK10. Together, these data suggest that half of the Nek family members (5 out of 11) and all of those so far tested in more detail are involved in the regulation of mitochondrial functional and morphological homeostasis (Figure 6).

## Data Availability

The original contributions presented in this study are included in the article. Further inquiries can be directed to the corresponding authors.
